# Retraction statement: Hanyu, Y., Hoshino, M., Usui, E., Sugiyama, T., Kanaji, Y., Hada, M., Nagamine, T., Nogami, K., Ueno, H., Sayama, K., Matsuda, K., Sakamoto, T., Yonetsu, T., Sasano, T., & Kakuta, T. (2023). Microvascular resistance reserve in the presence of functionally significant epicardial stenosis and changes after revascularization. Physiological Reports, 11, e15627

**DOI:** 10.14814/phy2.15830

**Published:** 2023-09-27

## Abstract

https://doi.org/10.14814/phy2.15627

The above article, published online on 10 March 2023 in Wiley Online Library (wileyonlinelibrary.com), has been retracted by agreement between the authors, the journal Editor‐in‐Chief, Josephine C. Adams, The Physiological Society and American Physiological Society, and John Wiley and Sons Ltd. The retraction has been agreed at the authors' request. Since publication, the authors have identified that the calculation of microcirculatory resistance does not require a correction factor. This invalidates several figures in the paper and the overall conclusions. The editors have no ethical concerns about the paper. The authors have prepared a Letter to the Editor (10.14814/phy2.15807) explaining, in detail, the error and their decision to retract the paper.

https://doi.org/10.14814/phy2.15627

The above article, published online on 10 March 2023 in Wiley Online Library (wileyonlinelibrary.com), has been retracted by agreement between the authors, the journal Editor‐in‐Chief, Josephine C. Adams, The Physiological Society and American Physiological Society, and John Wiley and Sons Ltd. The retraction has been agreed at the authors' request. Since publication, the authors have identified that the calculation of microcirculatory resistance does not require a correction factor. This invalidates several figures in the paper and the overall conclusions. The editors have no ethical concerns about the paper. The authors have prepared a Letter to the Editor (10.14814/phy2.15807) explaining, in detail, the error and their decision to retract the paper.

